# Non-Specific Protein Modifications by a Phytochemical Induce Heat Shock Response for Self-Defense

**DOI:** 10.1371/journal.pone.0058641

**Published:** 2013-03-11

**Authors:** Kohta Ohnishi, Shinya Ohkura, Erina Nakahata, Akari Ishisaka, Yoshichika Kawai, Junji Terao, Taiki Mori, Takeshi Ishii, Tsutomu Nakayama, Noriyuki Kioka, Shinya Matsumoto, Yasutaka Ikeda, Minoru Akiyama, Kazuhiro Irie, Akira Murakami

**Affiliations:** 1 Division of Food Science and Biotechnology, Graduate School of Agriculture, Kyoto University, Kyoto, Japan; 2 Department of Food Science, The University of Tokushima, Tokushima, Japan; 3 Department of Food and Nutritional Sciences, University of Shizuoka, Shizuoka, Japan; 4 Graduate School of Bioagricultural Sciences and School of Agricultural Sciences, Nagoya University, Nagoya, Japan; 5 Division of Applied Life Sciences, Graduate School of Agriculture, Kyoto University, Kyoto, Japan; 6 Department of Food and Nutrition, Kyoto Women's University, Kyoto, Japan; 7 Saga Nutraceuticals Research Institute, Otsuka Pharmaceutical Co., Ltd., Saga, Japan; Oak Ridge National Laboratory, United States of America

## Abstract

Accumulated evidence shows that some phytochemicals provide beneficial effects for human health. Recently, a number of mechanistic studies have revealed that direct interactions between phytochemicals and functional proteins play significant roles in exhibiting their bioactivities. However, their binding selectivities to biological molecules are considered to be lower due to their small and simple structures. In this study, we found that zerumbone, a bioactive sesquiterpene, binds to numerous proteins with little selectivity. Similar to heat-denatured proteins, zerumbone-modified proteins were recognized by heat shock protein 90, a constitutive molecular chaperone, leading to heat shock factor 1-dependent heat shock protein induction in hepa1c1c7 mouse hepatoma cells. Furthermore, oral administration of this phytochemical up-regulated heat shock protein expressions in the livers of Sprague-Dawley rats. Interestingly, pretreatment with zerumbone conferred a thermoresistant phenotype to hepa1c1c7 cells as well as to the nematode *Caenorhabditis elegans*. It is also important to note that several phytochemicals with higher hydrophobicity or electrophilicity, including phenethyl isothiocyanate and curcumin, markedly induced heat shock proteins, whereas most of the tested nutrients did not. These results suggest that non-specific protein modifications by xenobiotic phytochemicals cause mild proteostress, thereby inducing heat shock response and leading to potentiation of protein quality control systems. We considered these bioactivities to be xenohormesis, an adaptation mechanism against xenobiotic chemical stresses. Heat shock response by phytochemicals may be a fundamental mechanism underlying their various bioactivities.

## Introduction

All living organisms have substantial biological abilities to increase their resistance to various environmental stresses by inducing adaptation mechanisms. For example, plants biosynthesize polyphenols as antioxidants in response to UV light to mitigate oxidative damage, while mammals possess innate and adaptive immune systems for protection from xenobiotic stresses and microbial infections. In these phenomena, moderate stresses serve as driving forces to increase stress resistance, which is termed ‘hormesis,’ for beneficial effects provided by mild biological stress through adaptation mechanisms[1]. Plants are known to synthesize secondary metabolites, which consist of both constitutive and inducible chemicals for adapting to environmental stress[2]. On the other hand, there have been numerous studies on the bioactivities of food phytochemicals such as flavonoids and isothiocyanates, which have been reported to exhibit diverse physiological functions including anti-carcinogenesis activity[3], [4]. Recent mechanistic studies have also revealed that phytochemicals broadly disrupt multiple signal transduction pathways in mammals, which are associated with development of various diseases[3], [5]. However, fundamental questions related to why such compounds synthesized by plants for their own adaptation also regulate physiological functions in mammals remain to be answered. It is also important to note that the bioavailability of phytochemicals is substantially poor, because they are subject to biological inactivation through detoxification mechanisms. For example, anti-carcinogenic sulforaphane (SFN), an isothiocyanate broadly distributed among cruciferous vegetables, is immediately metabolized to its glutathione (GSH) conjugate by phase II detoxification enzymes and then excreted in urine[6]. This metabolic process is in contrast to that of glucose, for example, which is actively absorbed *via* its specific transporter. Interestingly, SFN is a potent inducer of phase II detoxification enzymes, which possess anti-oxidative properties[7]. Thus, xenobiotic phytochemicals reinforce mammalian self-defense systems, a phenomenon termed ‘xenohormesis’ by Howitz *et al.* [8].

Meanwhile, recent studies have revealed that phytochemicals specifically bind to proteins. In 2004, Tachibana *et al.* identified a 67-kDa laminin receptor as a receptor for (-)-epigallocatechin-3-gallate (EGCG), a major green tea polyphenol with chemopreventive potential[9]. Following that pioneering work, several studies of the target molecules of such as polyphenols and isothiocyanates (ITCs) have been presented. However, there are scant experimental data or insights regarding their binding selectivities. Considering that most, if not all, of these chemical structures are simple and small, it is reasonable to assume that they largely interact with proteins in a non-specific manner. In support of this notion, several ITCs including SFN were shown to bind to unidentified multiple proteins in cultured cells[10], [11], though the authors did not highlight those findings in their studies. Such non-selective bindings to proteins by xenobiotic chemicals are presumed to be potentially proteotoxic. However, there are no reports regarding the consequence of such non-specific protein modifications. On the other hand, it is well known that moderate proteotoxic stresses (*e.g.*, heat shock) are known to induce molecular chaperones including heat shock proteins (HSPs) as the major machinery of the protein quality control system[12]. Importantly, elevated expressions of HSPs may be associated with longevity[13] anti-diabetic[14] and anti-inflammatory[15] activities.

**Figure 1:**
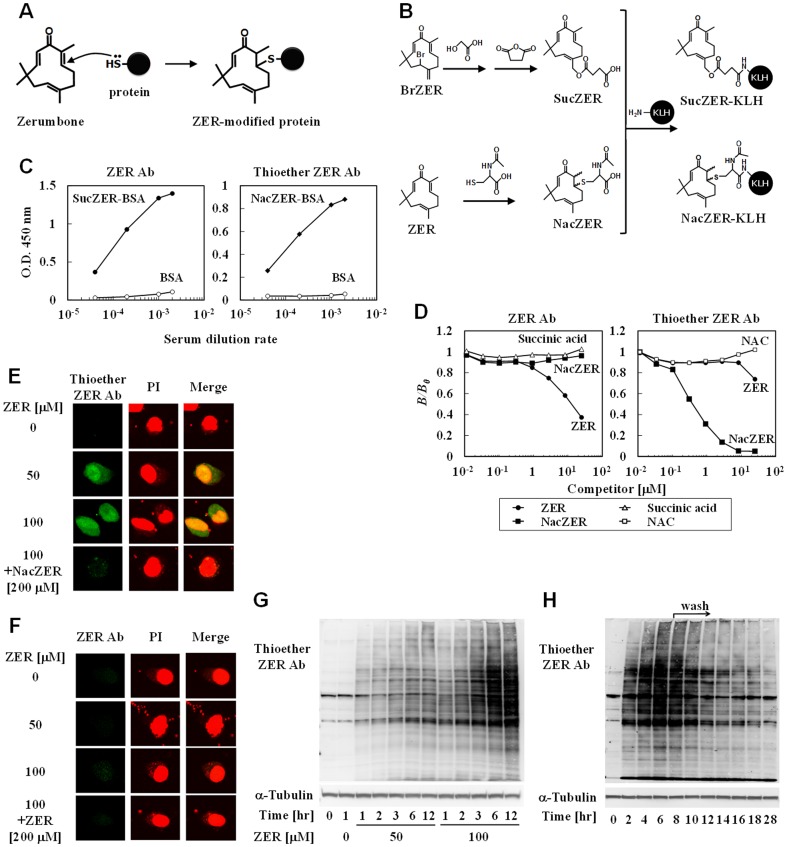
ZER non-selectively reacted with cellular proteins. (A) Nucleophilic addition of a protein thiol to zerumbone (ZER). (B) ZER was chemically derivatized to SucZER (succinylated ZER derivative) and NacZER (NAC-modified ZER derivative), followed by conjugation with KLH by carbodiimide procedure to generate Abs against ZER and its thiol adducts. (C) Generated polyclonal Ab (ZER Ab and thioether ZER Ab) titers were determined by ELISA, with each hapten moiety conjugated with BSA (SucZER-BSA and NacZER-BSA) or native BSA. These experiments were performed in duplicates. (D) Cross-reactivities of the Abs were determined by competitive ELISA. Competitors such as ZER, NacZER, succinic acid, and NAC (*N*-acetyl-L-cysteine) were added to hapten moiety conjugated BSA-coated wells with a primary Ab solution. The cross-reactivity of the antibody to the competitors was expressed as *B*/*B*_0_, in which *B* is the amount of antibody bound to the coating antigen in the presence of the competitor and *B*_0_ that in the absence of a competitor. These experiments were performed in duplicates. (E, F) Hepa1c1c7 cells were treated with ZER (0, 50, 100 µM) for 30 minutes, then fixed with 4% paraformaldehyde and permeabilized with 0.1% Triton X-100, followed by immunostaining with thioether ZER Ab (E) or ZER Ab (F). NacZER (E) or ZER (F) was added with the primary Ab as a competitor. Images showing cellular immunofluorescence (green: FITC, red: PI) were acquired using a confocal laser scanning microscope (original magnification: ×400). (G) Cells were treated with ZER (0, 50, 100 µM) for 1 minute to 12 hours, then lysed for western blot analysis using thioether ZER Ab. (H) Cells were treated with ZER (50 µM) for 0–6 hours, then washed with PBS and incubation in BioZER-free DMEM for another 2–22 hours. Cells were lysed for detection of modified proteins by western blot analysis with thioether ZER Ab.

We previously reported that zerumbone (ZER), a sesquiterpene present in *Zingiber zerumbet* Smith (Zingiberaceae) (Fig. 1A), exhibited cancer preventive[16], anti-inflammatory[17], and detoxifying[18] activities, though the mechanisms underlying these activities are not fully clarified. Our recent work with ZER-bound sepharose gel demonstrated covalent binding of an α,β-unsaturated carbonyl group in ZER to functional proteins *via* Michael addition[19] (Fig. 1A). Thus, we consider this small molecule to be a useful chemical tool to verify our hypothesis that non-specific protein modifications by phytochemicals activate protein quality control systems through induction of HSPs.

In the present study, we found that ZER binds to numerous proteins in hepa1c1c7 mouse hepatoma cells with low selectivity. ZER-modified proteins were recognized by HSP90 for inducing the expressions of multiple HSPs. Oral administration of ZER led to modifications of biomolecules and HSP70 induction in the livers of Sprague-Dawley (SD) rats, while ZER pretreatment conferred thermoresistance to hepa1c1c7 and *Caenorhabditis. elegans* (*C.elegans*). In addition, some phytochemicals, but not plant nutrients, were found to be notable HSP inducers. Based on our findings, we propose a hypothesis that non-specific bindings of phytochemicals induce activation of protein quality control systems, resulting in stress-resistant phenotypes.

## Materials and Methods

### Animals and Cells

Female BALB/c mice, 6 weeks of age, were purchased from Japan SLC (Shizuoka, Japan). All mice were quarantined for more than 1 week before starting the experiments, then kept under specific-pathogen free conditions. Mice were maintained in a controlled room at 24±2°C with a relative humidity of 60±5% and a 12-hour light/dark cycle. They were given commercial rodent pellets (MF: Oriental Yeast., Kyoto, Japan) and fresh tap water *ad libitum*, both of which were freshly exchanged twice a week.

Male SD rats were purchased from Japan SLC at 8 weeks of age and housed in a temperature-controlled (23±2°C) room for at least 2 weeks before beginning the present experiments. Rats were allowed free access to MF pellets and water. All rat experiments were done with male rats. The care and handling of the rats was in accordance with the Guidelines for Animal Care and Use of Otsuka Pharmaceutical Co., Ltd. The experimental protocol using rats have been approved by the animal experiment ethics committee of Otsuka Pharmaceutical Co., Ltd. (#2011-04).

Wild-type N2 nematodes were cultured using a standard *C. elegans* culturing method[20]. Age-synchronized populations were prepared as previously described[21]. Eggs were collected from gravid adult hermaphrodites using sodium hypochlorite and allowed to hatch at 25°C in S-basal buffer, which consisted of 100 mM NaCl and 50 mM potassium phosphate (pH 6.0). After hatching, the worms (L1-stage larvae) were transferred to fresh nematode growth medium (NGM) agar plates containing *Escherichia coli* OP50 at 25°C. All animal experiments using mice and nematodes were performed according to the Guidelines for the Regulation of Animals, as provided by the Experimentation Committee of Kyoto University, which approved the protocol of the present animal experiments (#21-44).

Hepa1c1c7, the hepatoma cells of mouse origin[22], were obtained from the American Type Culture Collection (Rockville, MD). The cells were grown in Dulbecco's modified Eagle's medium (DMEM) supplemented with 10% heat-inactivated fetal bovine serum (FBS), as well as penicillin (100 U/mL) and streptomycin (100 µg/mL) at 37°C under a humidified atmosphere of 95% air and 5% CO_2_.

### Reagents

Zerumbone (ZER) was purified as previously reported (purity >95%)[23]. Keyhole limpet hemocyanin (KLH), 1-ethyl-3-(3-dimethylaminopropyl) carbodiimide hydrochloride (EDC), and *N-*hydroxysuccinimide (NHS) were obtained from Pierce (Rockford, IL). DMEM and FBS were from Gibco BRL (Grand Island, NY). Lipopolysaccharide (LPS from *Escherichia coli* serotype 0127, B8) was purchased from Difco Labs (Detroit, MI). Antibodies (Abs) were obtained from the following sources: mouse anti-HSP90, mouse anti-HSP70, and rabbit anti-HSP40 Abs were from Enzo Biochem Inc. (New York, NY); mouse anti-α-tubulin Ab was from EMD Bioscience (La Jolla, CA); goat anti-β-actin Ab was from Santa Cruz Biotechnology (Santa Cruz, CA); rabbit anti-HSF1 and anti-rabbit Ab HRP-linked IgG Abs were from Cell Signaling Technology (Beverly, MA); and HRP-conjugated anti-mouse, HRP-conjugated anti-goat, and FITC-conjugated anti-mouse Abs were from DAKO (Tokyo, Japan). HRP-conjugated Streptavidin was purchased from Thermo Scientific (Waltham, MA). Bio-Adembeads Protein G was obtained from Ademtech SA (Pessac, France). Non-specific control small interfering RNA (siRNA) was purchased from Santa Cruz Biotechnology, and HSF1 siRNA and Lipofectamine^TM^ 2000 were from Invitrogen (Carlsbad, CA). All other chemicals were purchased from Wako Pure Chemical Industries (Osaka, Japan), unless otherwise specified.

### Synthesis of immunogens

ZER (1 g) was reacted with *N*-bromosuccinimide (1.1 eq) in 50% aqueous acetonitrile system at room temperature for 1 minute. 7-Bromo-2,9,9-trimethyl-6-methylenecycloundeca-2,10-dienone (BrZER) was purified by filtration. BrZER (50 mg) was reacted with glycolic acid (1.5 eq) and silver (I) oxide (2 eq) in diethyl ether at room temperature for 9 hours. The hydroxyl derivative of ZER was purified using thin-layer chromatography (TLC, ethyl acetate: *n*-hexane = 3:2) with TLC silica gel and a 60 F_254_ aluminium sheet (Merck Ltd., Tokyo, Japan). Subsequently, it was reacted with succinic anhydride (2 eq) in pyridine at room temperature for 40 hours to yield the succinylated ZER derivative (SucZER), which was purified on TLC (ethyl acetate: *n*-hexane = 2:1). Alternatively, ZER (250 mg) was reacted with *N*-acetyl-L-cysteine (2 eq) in 1N HCl and 50% aqueous acetonitrile at room temperature overnight. NacZER was purified on TLC (chloroform: methanol = 9:1) and subjected to a reverse phase high-performance liquid chromatography (HPLC) device equipped with a YMC-pack ODS-AQ (150×4.6 mm, YMC, Japan). HPLC was performed with 50% aqueous methanol containing 0.5% acetic acid as an eluent at a flow rate of 3 mL/minute with the UV detector set at 254 nm. Identification of the derivatives was performed by ^1^H-nuclear magnetic resonance (NMR) analysis using an ARX500 (Bruker, Japan) in CDCl_3_. SucZER and NacZER were separately coupled with carrier proteins using a carbodiimide procedure[24]. To prepare the conjugates of these haptens with proteins, they were activated by incubating with EDC (3 eq) and NHS (3 eq) in dimethylformamide (DMF), then added to KLH (10 mg/mL) and BSA (10 mg/mL) in phosphate-buffered saline (PBS) and incubated at room temperature for 4 hours. After incubation, the proteins were dialyzed in PBS at 4°C for 2 days. Protein concentrations were determined using a Bio-Rad Protein Assay Kit (Bio-Rad, Richmond, CA).

### Preparation of polyclonal antibodies

The obtained hapten-KLH conjugates (0.6 mg/mL in PBS) were emulsified with an equal volume of adjuvant. Six-week-old female BALB/c mice were separately immunized intraperitoneally with these emulsions (100 µL) and repeatedly boosted with immunogens (0.2 mg/mL) emulsified with an equal amount of adjuvant every 2 weeks. For the final boost, 100 µL of immunogens (0.5 mg/mL) without the adjuvant was intraperitoneally injected. Three days after the final boost, the mice were euthanized and serum samples were obtained. The antibodies were purified using a Melon™ Gel IgG Spin Purification Kit (Thermo Scientific, Waltham, MA).

### ELISA for determination of Ab titers

Native BSA and each hapten-BSA conjugate (5 µg/mL) in PBS were used to coat in 96-well plates, then incubated at 37°C for 1 hour. After washing 3 times with PBS containing 0.05% Tween 20 (TPBS), the wells were blocked with a 2% aqueous solution of Block Ace (GE healthcare, Buckinghamshire, UK) at 37°C for 1 hour. After washing, ZER and thioether ZER Abs (1∶500, 1000, 5000, 25000) in TPBS were added, and the wells were incubated at 37°C for 2 hours. After washing, HRP-conjugated anti-mouse IgG (1∶5000) was added and incubated at 37°C for 1 hour. The color developing reaction was performed by addition of TMB substrate solution (BD Biosciences Pharmingen, San Diego, CA). After stopping the reaction with 2N H_2_SO_4_ solution, binding of the antibody to the antigen was evaluated by measuring optical density at 450 nm.

### Competitive ELISA

For competitive ELISA, primary Abs were added to antigen-coated wells in the presence or absence of competitors. The competitive reactions were performed in PBS containing 0.5% DMSO at 37 °C for 90 minutes. Cross-reactivity of the Abs to the competitors was expressed as *B*/*B*_0_, in which *B* is the amount of antibody bound to the coating antigen in the presence of a competitor and *B*_0_ is that in the absence of a competitor.

### Immunostaining of intracellular ZER and its thiol-adducts

Hepa1c1c7 cells (2×10^4^/200 µL) were treated with ZER (0, 50, 100 µM) for 30 minutes. After washing, the cells were fixed for 20 minutes in PBS containing 4% paraformaldehyde. The membranes were permeabilized by exposing fixed cells to PBS containing 0.1% Triton X-100 for 30 minutes. The cells were then incubated in PBS containing 2% Block Ace for 1 hour and immunostained with ZER or thioether ZER Ab (1∶10) at 4°C overnight. They were then treated with FITC-conjugated anti-mouse Ab (1∶20) at room temperature for 1 hour and mounted with 50% glycerol in PBS. Images of cellular immunofluorescence were acquired using a MicroRadiance Confocal Laser Scanning microscope (Bio-Rad Laboratories). Propidium iodide (PI) (1 µg/mL) and RNase (100 µg/mL) were added to a secondary Ab solution for nuclear staining. Each competitor was added to the primary Ab solution and competitive assays were performed in PBS containing 0.5% DMSO.

### Western blot analysis

Hepa1c1c7 cells (1×10^5^/mL) were seeded into 24-well culture plates, and treated with the sample or vehicle (0.5% DMSO, *v/v*) for various times. Next, they were washed with PBS twice and lysed in lysis buffer [10 mM Tris, pH 7.4, 1% sodium dodecyl sulfate (SDS), 1 mM sodium metavanadate (V)], and centrifuged at 6000×*g* for 5 minutes. Denatured proteins were separated using SDS-polyacrylamide gel electrophoresis (PAGE) on 5% or 10% polyacrylamide gels, then transferred to Immobilon-P membranes (Millipore, Billerica, MA). After blocking with PBS containing 2% Block Ace for 1 hour, each membrane was treated with an appropriate specific primary Ab (1∶2000 or 200), followed by the corresponding HRP-conjugated secondary Ab (1∶2000). To detect BioZER-modified proteins, the membrane was treated with HRP-conjugated Streptavidin (1∶10000). The blots were developed using ECL Western blot detection reagents (GE Healthcare, Buckinghamshire, UK).

### Griess assay

NO production was determined based on the amount of nitrite, a stable end-product of NO. Nitrite concentrations in media supernatants were determined by use of a Griess reaction[25]. Hepa1c1c7 cells (1×10^5^/mL) were seeded into 24-well plates and pretreated with BioZER (0, 0.8, 4, 20 µM) for 30 minutes at 37°C, followed by exposure to LPS (100 ng/mL) for 24 hours. Each supernatant (100 µL) was mixed with 100 µL of Griess reagent [1% sulfanilamide in 5% phosphoric acid and 0.1% *N*-(1-naphthyl) ethylenediamine dihydrochloride in distilled water]. Absorbance was measured at 540 nm.

### Detection of cellular non-bound HSP90 recruitment

Hepa1c1c7 cells (1×10^6^/mL) were seeded into 60-mm dishes and washed with PBS twice, then lysed in native lysis buffer (10 mM BisTris, pH 7, 50 mM aminocaproic acid). Cell lysates were incubated with ZER (500 µM) for 8 hours, then incubated with non-treated lysates for 0, 2, 5, and 30 minutes. To immobilize generated protein complexes, the mixed lysates were treated with 0.05% glutaraldehyde in native lysis buffer and subjected to SDS-PAGE with 5% polyacrylamide gel for western blot analysis. Lysates pretreated with glutaraldehyde were used as a negative control and the lysates exposed to heat shock (incubation in 43°C waterbath) before glutaraldehyde treatment were used as a positive control.

### Immunoprecipitation of HSP90

Hepa1c1c7 cells (2×10^6^/10 mL) were seeded into 100-mm dishes and washed with PBS twice, then lysed in lysis buffer for immunoprecipitation [25 mM 3-(*N*-morpholino)propanesulfonic acid (MOPS), pH 7.5, 10% glycerol]. Cell lysates were treated with ZER (0, 100, 500 µM) for 8 hours and incubated with 0.2% anti-HSP90 Ab in the presence of Bio-Adembeads Protein G for 1 hour. The beads were washed twice with washing buffer [25 mM 3-(*N*-morpholino)propanesulfonic acid (MOPS), pH 7.5, 10% glycerol, 50 mM NaCl] and the immunoprecipitates were eluted using elution buffer [50 mM glycine, pH 2.7, 0.65% Tween 20].

### Determination of phosphorylated HSF1 (Ser326)

Hepa1c1c7 cells (1×10^6^/5 mL) were seeded into 60-mm dishes and treated with ZER (0, 100 µM) for 1 or 3 hours. After washing with PBS twice, cells were lysed, and lysates were subjected to a pSer^326^ HSF1 EIA Kit according to the manufacturer's instructions (Enzo Biochem Inc., New York, NY). Cells exposed to heat shock (incubation at 43°C in waterbath) for 15 minutes were used as a positive control.

### Real-time reverse transcription-polymerase chain reaction (qRT-PCR)

Hepa1c1c7 cells (1×10^5^/mL) were seeded into 24-well culture plates and treated with a sample or the vehicle (0.5% DMSO, *v/v*) for various times. Alternatively, D3 larvae nematodes were treated with ZER (0, 100, 200, 400 µM) for 62 hours or exposed to mild heat (incubation at 33°C in waterbath for 1 hour, followed by recovery at 25°C for 24 hours) in liquid NGM medium. Total RNA was isolated from cells and *C. elegans* using TRIzol reagent (Invitrogen, Carlsbad, CA), according to the manufacturer's specifications. The amount and purity of RNA were assessed by spectrophotometry using a SmartSpec® 3000 Spectrophotometer (Bio-Rad Laboratories). cDNA was synthesized using 1 µg of total RNA with an RNA PCR Kit (AMV). Thermal cycling was performed with a 7300 real time PCR system using SYBR green PCR mix (Applied Biosystems, Foster City, CA), according to the manufacturer's protocol. PCR conditions were as follows: 95°C for 3 minutes, 95°C for 10 seconds, and 60°C for 1 minute (mouse); 50°C for 2 minutes, 95°C for 2 minutes, 95°C for 15 seconds, 60°C for 30 seconds, and 72°C for 30 seconds (*C. elegans*). The primers and sequences used are summarized in Table 1.

**Table 1:** Primers used for RT-PCR.

Species	Gene	Primer	Sequence (5′ to 3′)
*Mouse*	*HSP90α*	Sense	AAAggCAggCTgACAAgA
		Antisense	AggggAggCATTTCTTCAgT
	*HSP90β*	Sense	gCggCAAAgACAAgAAAAAg
		Antisense	gAAgTggTCCTCCCAgTCAT
	*HSP70*	Sense	TggTgCTgACgAAgATgAAg
		Antisense	AggTCgAAgATgAgCACCgTT
	*HSP40*	Sense	TTACAAggCgAggAgAAgAC
		Antisense	TTgACAATCTgACCTggATg
	*CHOP*	Sense	AAgCCTggTATgAggATCTg
		Antisense	AgggTCAAgAgTAgTgAAgg
	*GRP78*	Sense	ACgCACTTggAATgACCCT
		Antisense	AATACgCCTCAgCAgTCTC
	*HPRT*	Sense	gTAATgATCAgTCAACggggAC
		Antisense	CCAgCAAgCTTgCAACCTTAACCA
*C.elegans*	*HSP-3*	Sense	CCgTCACCATCCAggTCTTC
		Antisense	ggTTCCCTTATCCTCggCAg
	*HSP-16.1*	Sense	gTCACTTTACCACTATTTCCgTCCAgCTCAACgTTC
		Antisense	CAACgggCgCTTgCTgAATTggAATAgATCTTCC
	*HSP-16.2*	Sense	CTgCAgAATCTCTCCATCTgAgTC
		Antisense	AgATTCgAAgCAACTgCACC
	*HSP-16.41*	Sense	ATTggggAgATTgTAAATgATg
		Antisense	gCgTTTCAAgTATCCATgTTCC
	*HSP-70*	Sense	gAAAggTTgAgATCCTCgCC
		Antisense	CATCgAAACgTCgTCCAATC
	*CDC42*	Sense	CTgCTggACAggAAgATTACg
		Antisense	CTCggACATTCTCgAATgAAg

### RNA interference (RNAi) of HSF1

Transfection of siRNA was performed using Lipofectamine™ 2000, according to the manufacturer's specifications. Briefly, 24 hours before transfection, cells (1.7×10^4^/mL) were seeded into 24-well plates. An siRNA solution (non-specific control, HSF1) was added to 50 µL of serum-free Opti-MEM I^®^ (1 µM final concentration). In another microtube, 2 µl of Lipofectamine™ 2000 was diluted in 50 µL of serum-free Opti-MEM I^®^. After adding the siRNA solution to the Lipofectamine™ 2000 solution, the transfection mixture was incubated for 25 minutes. This transfection mixture was then diluted in 500 µL of serum-free Opti-MEM I^®^, which was added to wells and cells were incubated for 6 hours. After replacing the medium with DMEM containing 10% FBS, the cells were incubated for another 24 hours. After washing twice with PBS, the cells were lysed and the lysates were subjected to western blot analysis, as described above.

### Thermotolerance test

Hepa1c1c7 cells (1×10^5^/mL) were seeded into 24-well plates and pretreated with ZER (0, 10, 25, 50 µM) for 24 hours at 37°C, or exposed to mild heat (incubation at 43°C in waterbath for 1 hour, followed by recovery at 37°C for 6 hours). Next, they were exposed to heat shock at 45°C in a waterbath for 1 hour. After recovery for 12 hours, cell viability was determined using a Cell Counting Kit-8 (WST-8, Dojindo Molecular Technology, Kumamoto, Japan). Briefly, after washing with PBS twice, cells were incubated in DMEM containing 5% WST for 1 hour and absorbance of the cell culture medium was measured at 450 nm. Values for cell viability of the positive control cells, which were not exposed to heat shock, were standardized as 100%.

D3 larvae nematodes (10 worms/200 µL) were treated with ZER (0, 100, 200, 400 µM) for 3 days or exposed to mild heat (incubation at 33°C in waterbath for 1 hour, followed by recovery at 25°C for 24 hours) in liquid NGM medium. Dead and alive nematodes were counted following exposure to heat shock at 37°C in a waterbath for 1 hour. The survival rate of the positive control nematodes, which were not exposed to heat shock, was standardized as 100%.

### Rat treatment

Twenty-four male SD rats (8 weeks old) were divided into control and experimental groups. Rats were orally administered ZER (0, 50 mg/kg) in corn oil twice a day for 1 week, then euthanized and the livers were collected. Livers were lysed in PBS or lysis buffer, and the lysates were subjected to competitive ELISA with thioether ZER Ab to determine the concentration of ZER-thiol adducts, or western blot analysis.

### Statistical analysis

Each experiment was performed at least 3 times and values are shown as the mean ± SD where applicable. Statistically significant differences between groups in each assay were determined using a Tukey-Kramer test, Dunnett's test, and Student's *t*-test,

## Results

### Global protein modifications by ZER

ZER is an electrophile that has potential reactivity with protein cysteine residues to form thiol ethers, as shown in our previous *in vitro* assay results (Fig. 1A)[19]. To detect interactions between ZER and proteins in cultured cells, we generated antibodies against ZER and its thiol ether derivative (Fig. 1B), termed ZER Ab and thioether ZER Ab, respectively (Fig. 1C). Competitive ELISA showed that the ZER Ab reacted with ZER, but not succinic acid or NacZER. Similarly, the thioether ZER Ab significantly recognized NacZER, but not *N*-acetyl-L-cysteine (NAC) or ZER (Fig. 1D). Next, hepa1c1c7 mouse hepatoma cells were treated with ZER (0, 50, 100 µM) for 30 minutes, then independently immunostained with each antibody. Interestingly, the ZER-thiol adducts were globally distributed throughout the cytoplasm and more abundantly in the nuclei (Fig. 1E). Co-treatment with NacZER (200 µM) and thioether ZER Ab abrogated that positive staining, validating the Ab specificity. In contrast, the intracellular localization of ZER was scarcely detectable (Fig. 1F). To verify protein modifications, ZER-treated cell lysates (0, 50, 100 µM cultured for 1–12 hours) were subjected to western blotting with thioether ZER Ab. ZER-modified proteins were detected within 1 hour after exposure, and then increased in both concentration- and time-dependent manners (Fig. 1G). It is also interesting to note that the modified proteins became dramatically undetectable after washing the cells, implying degradation through intracellular proteolysis mechanisms (Fig. 1H).

**Figure 2:**
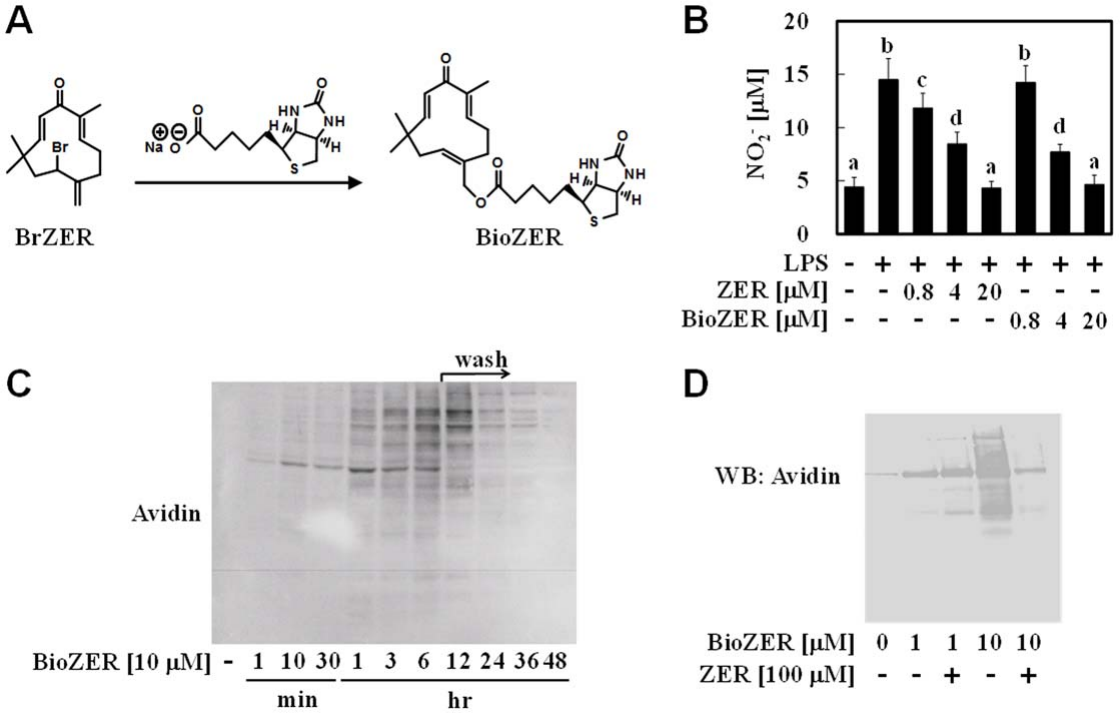
Biotin derivative of ZER was bound to numerous proteins with less selectivities. (A) BrZER was chemically reacted with biotin to yield the biotin derivative of ZER (BioZER). (B) Hepa1c1c7 cells were pretreated with the vehicle, ZER, or BioZER (0.8–20 µM), then exposed to LPS (100 ng/mL) for 24 hours. NO generation was quantified by measuring the concentration of nitrite using Griess reagent. The various characteristics were significantly different, as shown by Tukey-Kramer test results (*P*<0.05). This experiment was performed in triplicates. (C) Cells were treated with BioZER (10 µM) for 0 minutes to 6 hours, then washed with PBS and incubation in BioZER-free DMEM for another 1–42 hours. Cells were lysed for detection of modified proteins by western blot analysis with HRP-conjugated avidin. (D) Cells were co-treated with the vehicle or BioZER (1, 10 µM) with or without ZER (100 µM) for 30 minutes, then lysed for detection of modified proteins using western blot analysis with HRP-conjugated avidin.

Subsequently, we synthesized a biotin derivative of ZER (BioZER) to confirm its non-selective protein modifications (Fig. 2A). This chemical probe was designed to introduce a biotin ester at the opposite site of two α,β-unsaturated carbonyl groups, which are essential moieties of ZER to exhibit its bioactivity[18], [26]. First, we evaluated the suppressive activity of BioZER toward lipopolysaccharide (LPS)-induced nitric oxide (NO) generation in hepa1c1c7 cells. NO production was significantly reduced by pretreatment with BioZER in a concentration-dependent manner (Fig. 2B). Furthermore, the IC_50_ value of BioZER (9.1 βM) was comparable to that of ZER (12 µM), showing that BioZER can serve as a biological ZER mimic. We then attempted to detect BioZER-bound proteins in lysates from hepa1c1c7 cells by western blotting using HRP-conjugated avidin. Exposure of those cells to BioZER (10 µM) resulted in dramatic formation of BioZER-bound proteins (Fig. 2C), which disappeared with co-treatment with ZER (100 µM) (Fig. 2D). Thus, BioZER is a useful probe that competes with ZER for binding proteins. These findings obtained with anti-ZER Ab and BioZER revealed the non-specific binding property of ZER toward cellular proteins. BioZER-bound proteins became undetectable 18 hours after washing the cells (Fig. 2C), which is correspond to the decrease of ZER-modified proteins shown in Fig. 1H.

### ZER-modified proteins recognized by HSP90 for HSR

**Figure 3:**
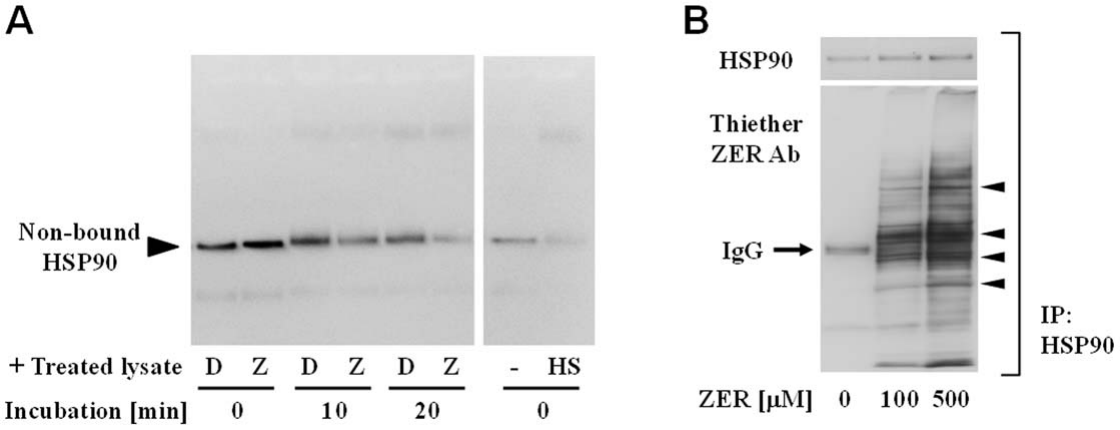
HSP90 was recruitedfor recognition of ZER-modified proteins. (A) Native hepa1c1c7 lysates from hepa1c1c7 cells were incubated with ZER (Z, 500 µM) or the vehicle (D, 0.5% DMSO, *v/v*) for 8 hours, then added to non-treated lysates. After 0–30 minutes, protein complexes were cross-linked with 0.05% glutaraldehyde for western blot analysis. Cells exposed to heat shock (incubation at 43°C in waterbath) served as a positive control (HS). (B) Cell lysates were treated with ZER (0, 100, 500 µM) for 8 hours, then incubated with anti-HSP90 Ab and Bio-Adembeads Protein G for 1 hour. Immunoprecipitates were eluted and subjected to western blot analysis. Arrows show significantly increased bands.

**Figure 4:**
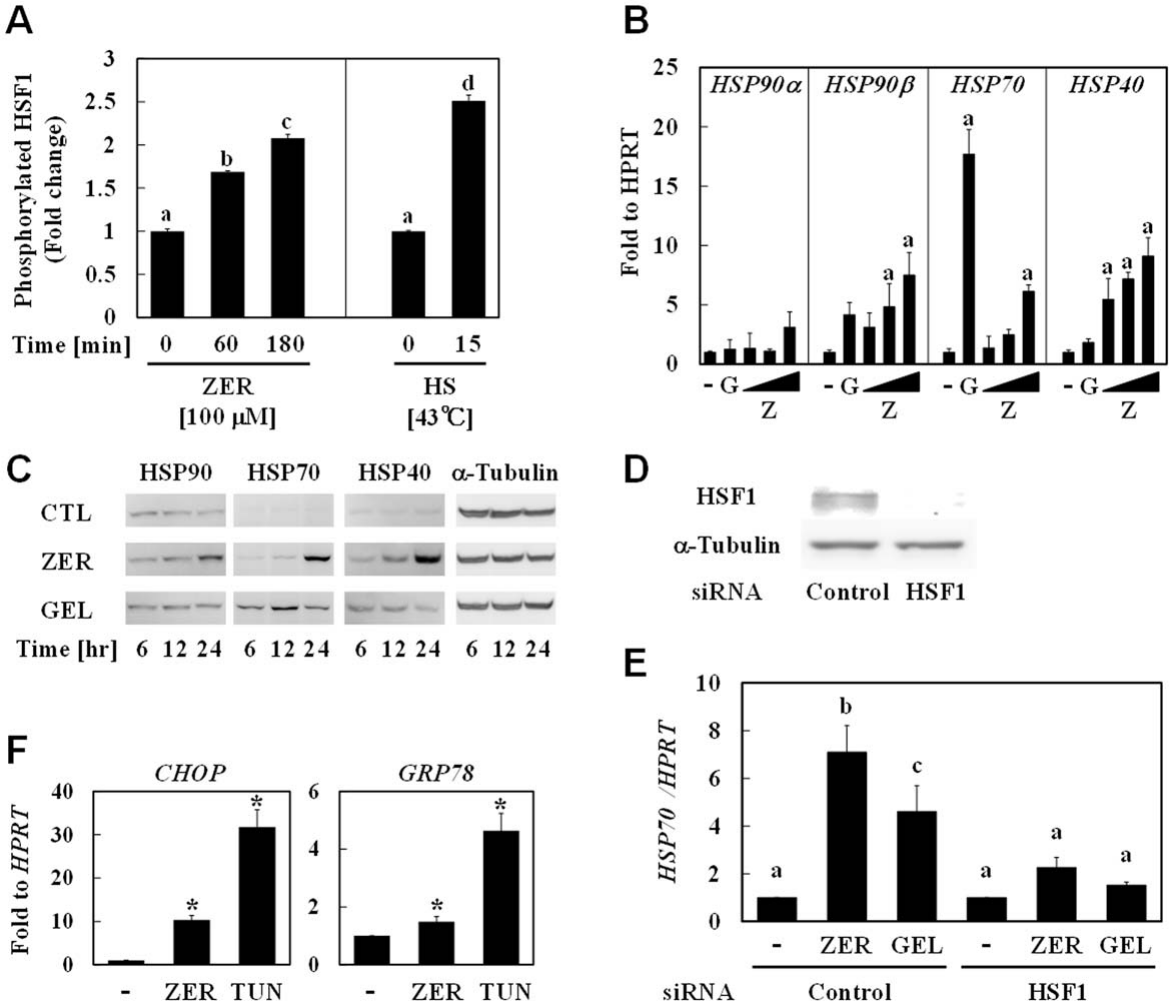
ZER induced HSPs expressions through HSF1 activation. (A) Hepa1c1c7 cells were treated with ZER (100 µM) for 1 or 3 hours. HSF1 phosphorylated at Ser326 in cell lysates was detected by ELISA. As a positive control, cells were exposed to heat shock (HS; incubation at 43°C in waterbath) for 15 minutes and examined. This experiment was performed in quadruplicates. (B) Cells were treated with the vehicle, ZER (Z; 10, 25, 50 µM) or geldanamycin (G; 1 µM) for 3 hours, then total RNA was subjected to qRT-PCR to semi-quantify the expressions of HSP90α, HSP90β, HSP70, and HSP40. HPRT expressions were also measured as internal standards. This experiment was performed in triplicates. a, versus CTL by Dunnett's test (*P*<0.05). (C) Cells were treated with the vehicle, ZER (50 µM), or geldanamycin (GEL; 1 µM) for 6-24 hours, then lysed for western blot analysis. (D) Cells were treated with Lipofectamine™ 2000 and a siRNA solution (control and HSF1, 75 nM) for 6 hours. The culture medium was replaced with DMEM containing 10% FBS and incubated for another 24 hours. Cell lysates were subjected to western blot analysis. (E) Cells were treated with Lipofectamine™ 2000 and a siRNA solution (control and HSF1, 75 nM) for 6 hours, then the culture medium was replaced with DMEM containing 10% FBS and incubation was performed for another 24 hours. Then, siRNA-transfected cells were treated with the vehicle, ZER (50 µM), or geldanamycin (GEL; 1 µM) for 15 hours, and total RNA was subjected to qRT-PCR to semi-quantify the expression of HSP70. HPRT expression was also determined as an internal standard. This experiment was performed in quadruplicates. The various characteristics were significantly different, as shown by Tukey-Kramer test result (*P*<0.05). (F) Cells were treated with the vehicle, ZER (50 µM) or tunicamycin (TUN; 1 µM) for 6 hours, then total RNA was subjected to qRT-PCR to semi-quantify the expressions of CHOP and GRP78. HPRT expressions were also measured as internal standards. This experiment was performed in triplicates. *P<0.05 vs. DMSO by Dunnett's test.

HSP90, a major constitutive molecular chaperone, has a biochemical property allowing it to bind to a hydrophobic region of denatured proteins and is also a negative regulator of the transcription factor HSF1, which regulates the expressions of inducible isoforms of HSPs such as HSP70 and HSP40[27]. Upon exposure to stress, denatured proteins induce non-bound HSP90recruitement, which enables HSF1 to transactivate the target genes. Thus, we speculated that ZER-modified proteins, like denatured ones, are recognized by HSP90 for HSR. To test this hypothesis, we examined whether HSP90 bound to ZER-modified proteins using *in vitro* assays. When a non-treated hepa1c1c7 cell lysate was added to another lysate containing ZER-modified proteins, the amount of non-bound HSP90 protein was dramatically decreased after 10 and 20 minutes, with a more distinct decrease seen with lysates immediately exposed to heat shock at 43°C (Fig. 3A). Collectively, non-bound HSP90 may be recruited for recognition of and binding to ZER-modified proteins, although such HSP90 complexes with higher molecular sizes were not detected.Additionally, co-immunoprecipitation with anti-HSP90 Ab suggested the existence of biochemical interactions between modified proteins and HSP90 (Fig. 3B). Taken together, HSP90 is suggested to recognize ZER-bound proteins. To verify HSR after HSP90 recognition of ZER-modified proteins, we evaluated HSF1 phosphorylation at Ser326, one of the residues crucial for its full activation[28]. Hepa1c1c7 cells were treated with ZER (100 µM), and the levels of phosphorylated HSF1 (Ser326) were semi-quantified by ELISA. As shown in Figure 4A, ZER induced HSF1 phosphorylation in a time-dependent manner. Consistent with this observation, treatment with ZER (0–50 µM) resulted in concentration-dependent increases of mRNA expressions of HSP90β, HSP70, and HSP40, but not HSP90α (Fig. 4B). Geldanamycin, a specific inhibitor of HSP90 used as a positive control, up-regulated HSP90β and HSP70 expressions. In support of these findings, ZER induced dramatic increases in HSP70 and HSP40 protein levels (Fig. 4C). Subsequently, we down-regulated the expression of HSF1 by siRNA to verify whether the HSP-inducing activity of ZER was dependent on HSF1 (Fig. 4D). HSF1 silencing abolished HSP70 induction by ZER, with similar results seen following geldanamycin treatment (Fig. 4E). Additionally, we investigated the possibility of ZER for inducing unfolded protein response, since ZER might modify global proteins (Fig. 1E) even in endoplasmic reticula (ER). ZER (50 µM) or tunicamycin (1 µM), a glycosylation inhibitor used as a positive control, up-regulated mRNA expressions of CHOP (C/EBP-homologous protein) and GRP78 (glucose-regulated protein 78), both of which are known as ER stress markers (Fig. 4F).

### ZER confers thermoresistant phenotype

**Figure 5:**
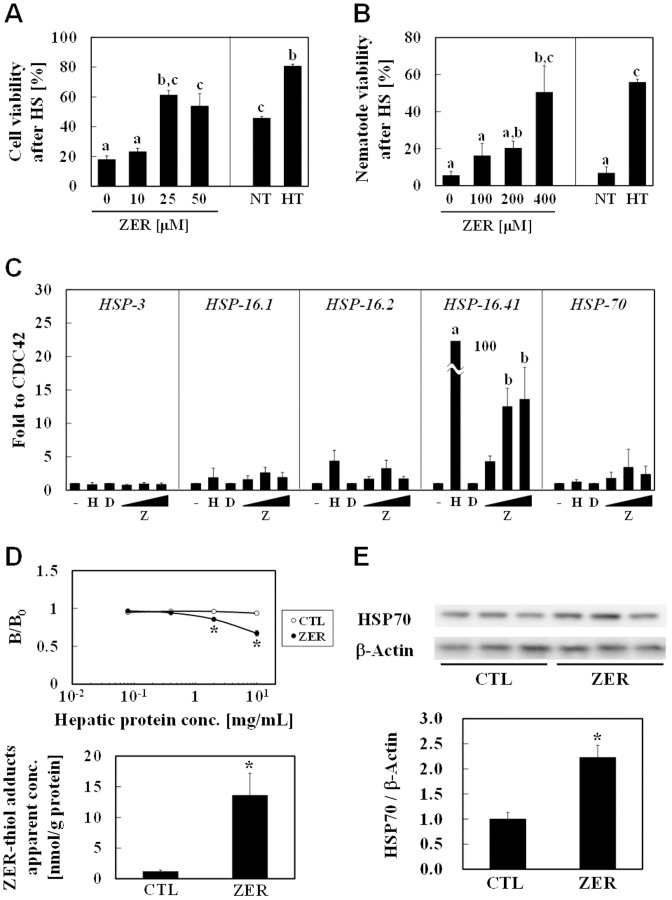
ZER induced HSPs expressions and conferred thermoresistant phenotype *in vivo*. (A) Hepa1c1c7 cells were treated with ZER (0, 10, 25, 50 µM) for 24 hours or exposed to mild heat (43°C) for 1 hour (HT), followed by recovery at 37°C for 6 hours. Cells were then exposed to heat shock (45°C) for 1 hour. After another 12 hours of incubation, cell viability was determined using a WST-8 test. Non-treated cells were used as a negative control (NT). This experiment was performed in triplicates. (B) D3 nematode larvae were pretreated with ZER (100, 200, 400 µM) for 3 days or pre-exposed to mild heat (33°C) for 1 hour (HT), followed by recovery at 25°C for 24 hours. After exposure to heat shock (incubation in 37°C waterbath) for 1 hour, survival rate was determined. Non-treated nematodes (NT) were used as a negative control. This experiment was performed in quadruplicates. The various characteristics were significantly different, as shown by Tukey-Kramer test results (*P*<0.05) (A, B). (C) D3 larvae nematodes were treated with ZER (Z; 100–400 µM) or the vehicle (D; 0.5% DMSO, *v/v*) for 62 hours, or exposed to mild heat (33°C) for 1 hour (H), followed by recovery at 25°C for 24 hours. HSPs expressions were semi-quantified by qRT-PCR. CDC42 expressions were also measured as internal standards. a, versus CTL; b, versus DMSO by Dunnett's test (*P*<0.05). This experiment was performed in quintuplicate. (D) SD rats (8 weeks old) were orally administered ZER (50 mg/kg) or the vehicle (CTL, corn oil) twice a day for 1 week, then euthanized and the livers were collected. Those were lysed with PBS, then lysates were added to NacZER-BSA-coated ELISA places as a competitor with a thioether ZER Ab solution. The amount of ZER-thiol adducts in liver lysates was semi-quantified based on a calibration curve using NacZER. This experiment was performed in triplicates. (E) Livers lysates were also subjected to western blot analysis for HSP70 determination. This experiment was performed in sextuplicate.*P<0.05 vs. CTL by Student's *t* test (D, E).

Increased HSP levels have been well documented to confer thermoresistance[29]. Therefore, phenotypic changes of both hepa1c1c7 cells and *C. elegans* pretreated with ZER were assessed by thermotolerance tests. ZER at concentrations of 25 and 50 µM markedly suppressed heat shock-induced cytotoxicity in hepa1c1c7 cells. Mild heat exposure at 43°C for 1 hour also conferred significant thermoresistance (Fig. 5A). On the other hand, treatment of nematodes with heat shock at 37°C for 1 hour resulted in a substantial decrease of the survival rates down to 10% or less. However, ZER administration (200 and 400 µM) for 3 days significantly increased those rates (Fig. 5B) and those effects were comparable to that seen with mild heat pretreatment at 33°C for 1 hour. Next, mRNA expression levels of several HSPs were quantified by qRT-PCR (Fig. 5C). The expression level of HSP3, a constitutive HSP, was unchanged following heat shock and ZER treatment. Interestingly, both heat shock and ZER markedly increased the expression of HSP16.41, which is highly expressed in the intestines and pharynx[30]. We then attempted to detect ZER-modified molecules in livers of ZER-administered rats by competitive ELISA with thioether ZER Ab. The apparent concentration of ZER-thiol adducts was determined to be 12 nmol/g of liver protein (Fig. 5D). Consistent with this finding, ZER significantly induced the expression level of HSP70 protein as compared with the vehicle control (Fig. 5E). These results demonstrated that this compound is orally active for modifying biomolecules and thereby induces HSP70 expression in rat livers.

### Several plant secondary metabolites, but not nutrients, induce HSP70

**Figure 6:**
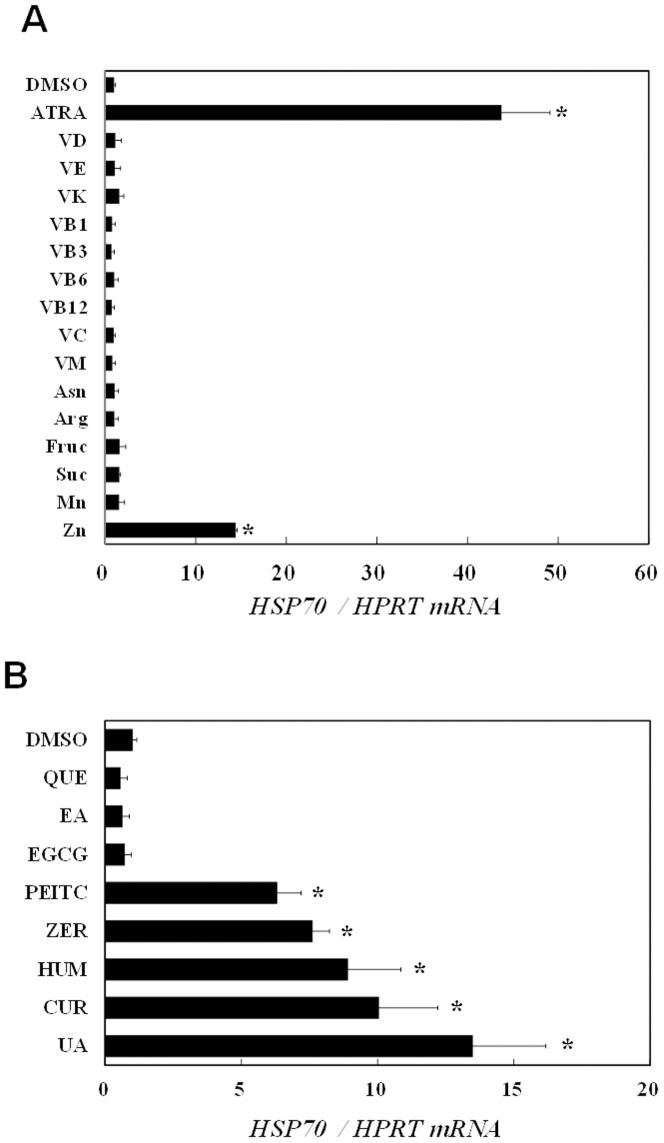
Screening of food ingredients for HSP70 inducing activities. Hepa1c1c7 cells were treated with the vehicle, nutrients such as all-*trans* retinol (ATRA; vitamin A), ergocalciferol (VD; vitamin D), α-tocopherol (VE; vitamin E), phylloquinone (VK; vitamin K), thiamin (VB1; vitamin B_1_), nicotinic acid (VB3; vitamin B_3_), pyridoxine (VB6; vitamin B_6_), cyanocobalamin (VB12; vitamin B_12_), ascorbic acid (VC; vitamin C), folic acid (VM; vitamin M), asparagine (Asn), arginine (Arg), fructose (Fruc), sucrose (Suc), manganese (II) sulfate (Mn), and zinc chloride (Zn) (A) or phytochemicals such as quercetin (QUE), ellagic acid (EA), (-)-epigallocatechin-3-gallate (EGCG), phenethyl isothiocyanate (PEITC), zerumbone (ZER), α-humulene (HUM), curcumin (CUR), and ursolic acid (UA) (B) for 6 hours, then total RNA was subjected to qRT-PCR to semi-quantify HSP70 expression. HPRT expressions were also measured as internal standards. All compounds were treated at their nonlethal and maximum concentrations (Mn: 0.016 µM, VD: 10 µM, VB1, VB3, VB6, Asn, and Arg: 500 µM, PEITC: 2 µM, HUM: 25 µM, all others treated at 50 µM). These experiments were performed in triplicates. *P<0.05 vs. DMSO by Dunnett's test (A, B).

Finally, we evaluated the HSP70 inducing activities of various natural compounds including plant primary and secondary metabolites to examine whether this activity is limited to that seen with ZER. Hepa1c1c7 cells were exposed to each compound at nonlethal, maximum concentrations for 6 hours, then HSP70 mRNA expression was determined by qRT-PCR. Among the tested nutrients, only all-*trans* retinol (ATRA) and zinc chloride showed potent activities (Fig. 6A), implying that most nutrients are not recognized as xenobiotic stressors, because they are essential for mammalian homeostasis. Next, we examined HSP70 induction by phytochemicals, including polyphenols and terpenoids (Fig. 6B). It is interesting to note that most polyphenols examined in this study did not up-regulate HSP70, but rather tended to suppress it, whereas hydrophobic phytochemicals such as α-humulene and ursolic acid markedly induced HSP70. Furthermore, electrophilic compounds, including phenethyl isothiocyanate and curcumin, were also identified as potent HSP70 inducers. These results support our hypothesis that non-specific protein modifications induce HSR, because both hydrophobicity and electrophilicity are major determinants for the non-specific interactions of chemicals with proteins.

## Discussion

In the present study, we attempted to uncover the biological consequences of non-specific protein modifications by phytochemicals. We anticipated that ZER, an electrophilic small molecule, would have less binding selectivity to cellular nucleophiles, which was shown by findings regarding its non-selective protein modifications (Fig. 1G, 2C). On the other hand, we observed only scant positive immunostaining by ZER Ab (Fig. 1F). Although we cannot rule out the possibility that it might be due to poor titer of ZER Ab (Fig. 1D), ZER may be rapidly conjugated with biological thiol groups. Given that the intracellular concentration of GSH, a nucleophilic tripeptide, is around 10 mM in liver cells[31], these results imply that this compound rapidly binds to not only proteins, but also to GSH. In support of this notion, we previously showed that ZER treatment reduced GSH concentration in a cellular system[18]. SFN, another electrophilic phytochemical, was also reported to be predominantly conjugated to GSH in hepa1c1c7 cells[32]. Considering that GSH plays a pivotal role for detoxification of electrophilic xenobiotics, it is reasonable that xenobiotic ZER was conjugated to it for excretion. Therefore, ZER-thiol adducts in hepa1c1c7 cells (Fig. 1E) and rat livers (Fig. 5D) may, at least in part, consist of both protein and GSH adducts.

HSPs participate in the protein quality control system by binding to hydrophobic regions in denatured proteins, which contributes to prevention of their aggregation and repairs their unfolding structures. HSP90, a major constitutive isoform, is known to repress the expression of inducible HSPs by sequestering HSF1 under a homeostatic condition[33]. HSP90 binds to denaturing proteins only when cells are exposed to denaturing stresses and thereby dissociates from HSF1 for HSR induction. Our results (Fig. 3A, B) suggest that proteins non-selectively modified by ZER may serve as clients for HSP90. Other electrophiles, such as 4-hydroxy-2-nonenal, an endogenous lipid peroxide, and SFN were previously reported to induce HSR in cultured cells[34], [35]. However, it is worth noting that the mechanisms underlying those actions were shown to be only specific binding to HSP90, similar to that of geldanamycin, which targets the ATP-binding pocket. In addition, 6-methylsulfinylhexyl isothiocyanate was recently shown to modify recombinant human HSP90 at Cys521 *in vitro*, while it was bound to numerous cellular proteins with less selectivity[11]. To the best of our knowledge, this is the first study to demonstrate non-specific protein modifications by the chemical trigger HSR. Essential questions regarding how the on- and off-target effects of those electrophiles contribute to their HSRs remain to be answered. On the other hand, ZER up-regulated the expressions of ER stress markers (Fig. 4F), showing that it also induces unfolded protein response. Although the mechanism underlying ER stress induction is not clarified, ZER might modify even ER proteins, which is supported by the data on intracellular global distribution of ZER-thiol adducts (Fig. 1E).

We demonstrated that ZER confers a thermoresistant phenotype to hepa1c1c7 cells and *C. elegans* (Fig. 5A, B). Importantly, Van *et al.* showed that HSP expression levels were related to this phenotype in H35 rat hepatoma cells[36]. On the other hand, it should be noted that thermoresistance is considered to be conferred not only by amplified protein refolding systems, but also by proteolysis machineries. Although the biological contribution of proteolysis to ZER-increased thermoresistance remains to be clarified, we found that ZER- or BioZER-modified proteins markedly disappeared through unknown mechanisms (Fig. 1H, 2C). In most proteolysis processes, unfolding proteins recognized by HSPs are ubiquitinated by CHIP[37], an E3 ubiquitin ligase, and then degraded by a proteasome or lysosome. Moreover, HSP70 induction has been shown to enhance autophagy in mesothelial cells[38]. Thus, putative proteolysis activation by ZER might also contribute to thermoresistance through HSP induction. In support of this speculation, tagging by small and hydrophobic molecules was recently reported to promote proteolysis in living cells[39]. Iwata *et al.* also demonstrated that proteolysis activity in the nucleus is lower than that in cytoplasm[40]. These observations may be consistent with our findings that ZER-thiol adducts were detected more abundantly in the nucleus than cytoplasm (Fig. 1E). Taken together, ZER-modified proteins might be degraded through the above-mentioned proteolysis mechanisms for homeostasis, an issue that is now being investigated in our laboratory.

The present is the first study to widely screen plant food ingredients for their ability to induce HSP70. Our screening test results strongly support our hypothesis that xenobiotic phytochemicals have potential for inducing HSR by providing mild proteostress. Plant secondary metabolites with higher hydrophobicity or electrophilicity, such as phenethyl isothiocyanate, α-humulene, curcumin, and ursolic acid, showed potent HSP70 inducing activities, whereas all of the examined polyphenols were virtually inactive. These contrasting results support the notion that both electrophilicity and hydrophobicity are fundamental properties of phytochemicals to induce HSR presumably through non-specific protein interactions. Considering that HSR is induced by HSP90 recognition of hydrophobic surfaces exposed in denatured proteins, hydrophobicity of phytochemicals may be a crucial structural determinant for their HSP70 inducing activities.

Consistent with our present results, EGCG and quercetin were shown to suppress HSP70 expression in other cell lines[41], [42]. On the other hand, most nutrients including vitamins, amino acids, saccharides, and metals were not found to be active, except for ATRA and zinc (Fig. 6A). ATRA has been reported to induce small HSP through the activation of RAR/RXR, its specific nuclear receptor[43], which is apparently a different mechanism from that of non-specific protein interactions. Our findings for zinc are consistent with those reported by Hatayama *et al.*, who showed that it activated HSF1 in FM3A cells[44]. Since zinc cation is well known to have a fundamental role in protein folding through coordinate bonds with some amino acid residues, it might induce HSR through modulation of protein conformations. Meanwhile, it is intriguing to ask why nutrients with high hydrophobicity, such as vitamins D, E, and K, did not induce HSP70 in our study, as their ClogP values (9.7–12.3) are considerably higher than those of active phytochemicals (2.3–6.8). One explanation for this contradiction may be related to the fact that those lipophilic vitamins preferentially bind to their specific receptors and binding proteins.

Hormesis is a beneficial effect provided by mild biological stress through adaptation mechanisms[1]. It is important to note that xenohormesis, a hormesis effect caused by xenobiotic chemicals, has been shown to exhibit significant physiological functions. For example, previous findings showed that induction of anti-oxidant and detoxifying enzymes by cruciferous phytochemicals may contribute to their chemopreventive effects[45]. Also, ZER induced heme oxygenase-1 (HSP32) and thereby conferred oxidative stress resistance to hepatocytes[18]. In the present study, we revealed protein modification-mediated thermoresistance as a xenohormesis effect of ZER, which warrants further investigation of its roles in other beneficial effects. On the other hand, xenohormesis can only be achieved when stress is under the limitation of adaptive capacity. An overdose of phytochemicals should cause a breakdown of self-defense systems, which would exhibit toxic effects. For example, in our recent study we found that oral administration of green tea polyphenols at a high dose aggravated DSS-induced acute colitis in ICR mice[46]. β-Carotene, a representative carotenoid broadly present in vegetables, has also been reported to increase lung cancer risk in smokers when given at a high dose[47]. Conversely, Massie *et al*. reported an interesting finding that sodium azide, a toxic compound used as antiseptic agent, induced HSP expression and conferred thermoresistance to *C. elegans* at low, but not high, doses[48]. Such beneficial effect of a so-called ‘poisonous compound’ well illustrates that hormesis and toxic effects induced by chemicals are essentially dependent on their dosages.

**Figure 7:**
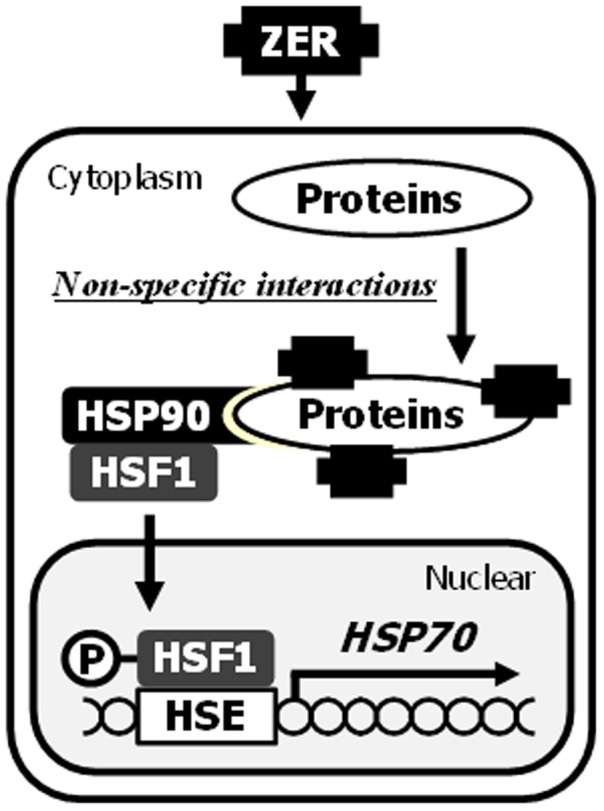
Proposed mechanisms underlying HSP70 induction by ZER. Xenobiotic phytochemicals like ZER are bound to numerous cellular proteins with less selectivities, which are recognized as proteostress by HSP90. Subsequently, HSF1 was dissociated from HSP90 and activated for HSPs induction.

In conclusion, our findings show activation of a protein quality control system by non-specific chemical protein modifications. ZER bound to numerous proteins in both cultured hepatocytes and rat livers, and those modified proteins were recognized by HSP90 for HSR induction (Fig. 7). Importantly, various plant secondary metabolites showed significant HSP70 induction activities. Thus, daily intake of phytochemicals might be described as a type ‘chemical training’ consisting of repetitive and chronic chemical stress exposure and adaption.
